# Effects of photobiomodulation associated with vestibular rehabilitation in the treatment of patients with vestibular symptoms and tinnitus: a triple-blind randomized clinical trial

**DOI:** 10.1590/2317-1782/e20230305en

**Published:** 2026-02-09

**Authors:** Thales Roges Vanderlei de Góes, Marine Raquel Diniz da Rosa, Giorvan Anderson dos Santos Alves, Rafael Nóbrega Bandeira, Sávio Bastos

**Affiliations:** 1 Departamento de Fonoaudiologia, Universidade Federal da Paraíba – UFPB - João Pessoa (PB), Brazil.

**Keywords:** Vestibular Diseases, Low Intensity Light Therapy, Postural Balance, Tinnitus

## Abstract

**Purpose:**

To analyze the effects of photobiomodulation (PBM) associated with vestibular rehabilitation (VR) in adults with vestibular symptoms and tinnitus, compared to VR combined with placebo PBM, regarding the reduction of tinnitus discomfort and improvements in vestibular function, postural balance, and dynamic visual acuity.

**Methods:**

Randomized, triple-blind clinical trial with 20 volunteers diagnosed with vestibular hypofunction and chronic tinnitus. Participants were allocated into two groups: research group (RG), which received VR combined with active PBM; and control group (CG), which received VR combined with placebo PBM. Both groups underwent ten intervention sessions, twice a week, over five weeks. Pre- and post-intervention assessments included: Tinnitus Handicap Inventory (THI), Dizziness Handicap Inventory (DHI), Visual Analog Scale (VAS), dynamic visual acuity (DVA) test, and posturography. Data were analyzed using Student's t-test and the Mann-Whitney test (p<0.05).

**Results:**

In the post-intervention comparison between groups, the RG showed significant improvement in VAS for tinnitus (p=0.046), and in the functional (p=0.003), emotional (p=0.002), and total (p=0.000) scores of the THI. The Sensory Organization Test parameter showed a significant pre- and post-treatment difference (p=0.003) only in the RG. VAS for vestibular symptoms, DHI scores, and the Composite Equilibrium Score improved in both groups, with no significant difference between them.

**Conclusion:**

PBM combined with VR significantly reduced tinnitus discomfort and showed superior performance in vestibular function analysis. VR alone did not demonstrate any effect on tinnitus reduction.

## INTRODUCTION

Vestibular symptoms (VSs) result from an imbalance in information processing by the vestibular, visual, and somatosensory systems. Dysfunction can occur due to alterations in one or more systems^(1)^. Therefore, they are caused by a variety of etiologies^(2-5)^.

The term vertigo should be considered within the definition of vestibular symptoms, referring to the distorted sensation of self-movement occurring during or in the absence of head movement^(2)^, while dizziness can be considered as a sensation of spatial disorientation^(2)^. Vestibulovisual and postural symptoms can also be included in addition to these VSs^(2)^.

The success of pharmacological measures in VSs depends on an accurate diagnosis, treatment with the appropriate medication, at the appropriate dosage and duration. Inadequate choices can render treatment ineffective^(2,5)^. Another therapeutic option for VSs is Vestibular Rehabilitation (VR), a physiological treatment form which considers symptoms and functional changes. VR can be defined as a set of therapeutic procedures aimed at promoting plasticity of the central nervous system through adaptation, compensation, and vestibular substitution exercises^(2,3,6,7)^. There is no single rehabilitation model; the therapeutic plan must be tailored to the individual’s specific VSs^(3,7-9)^. Systematic reviews with meta-analysis^(8,9)^ and guidelines^(2,3)^ demonstrate moderate to strong evidence of the efficacy and safety of VR in unilateral or bilateral vestibular hypofunction.

In turn, tinnitus can be defined as the conscious perception of a sound in the absence of an external auditory stimulus^(10)^. It is classified as subjective in the vast majority of cases, meaning only the affected individual can hear the sound^(10)^. The prevalence of tinnitus increases with age; approximately 9.7% of the global population of young adults between 18 and 44 years of age perceive tinnitus, a rate which increases to 23.6% in people over 65 years of age. The combined prevalence of severe tinnitus (meaning tinnitus that reports discomfort) among adults is estimated at 2.3%^(11)^. Although its pathophysiology is multifactorial and controversial, tinnitus could result from abnormal neural activity at any level of the auditory system^(12)^.

Tinnitus and VSs can occur simultaneously or independently^(13)^. The combination of symptoms is very common in the aging process^(14)^, vestibular migraine^(15)^, Ménière's syndrome^(16)^, and emotional disorders^(17)^. Both impair the individual’s quality of life, which can limit daily activities^(14-17)^.

Like the origin of tinnitus, treatments vary^(18)^. When there is an identifiable cause, it must be treated. Controlling the cause may not be sufficient to reduce or eliminate tinnitus^(13)^. Several therapeutic possibilities with varying degrees of success exist, such as Auditory Counseling, Retraining Therapy (RTT), Sound Masking, the use of Hearing Aids, Behavioral Therapy, Mindfulness, Manual Therapy, and Photobiomodulation (PBM)^18)^.

PBM therapy is a form of light therapy which uses non-ionizing light sources, such as lasers and light-emitting diodes (LEDs) that act as blood microcirculation facilitators through sympathetic neural inhibition. This increases cell proliferation, resulting in easier synthesis of adenosine triphosphate (ATP) in mitochondria^(18,19)^. This process accelerates repair and reduces damage to irradiated cells and tissues^(20)^.

The clinical outcomes of PBM’s effects on tinnitus remain controversial. Systematic reviews^(18,19)^ have analyzed studies with positive effects on symptom perception and discomfort, but others did not differentiate between the two groups. This discrepancy may be caused by differences in the irradiation methods used or by differences in patient samples. Regarding the use of PBM in VSs, a dose of gentamicin was injected into male, 12-week-old mice to induce vestibulopathy in an animal model study^(21)^. PBM was performed on seven consecutive days. The treatment normalized the vestibule (measured using a vestibular function test which evaluated the vestibulo-ocular reflex) and returned the cupula histology to near-normal, while the vestibule remained compromised in the control group. This demonstrates that in addition to the treatment being non-invasive and without reported adverse effects, it may be a promising resource for vestibular alterations in humans.

From this perspective, VR and PBM could be complementary therapies for VSs and tinnitus. The coexistence of these symptoms can be attributed to common neurophysiological mechanisms, in which vestibular dysfunction and abnormal neural activity in the auditory system interrelate, exacerbating the patient’s experience^(13)^. By focusing on functional rehabilitation^(2)^, VR aims to restore postural stability and vestibular functionality through specific exercises that promote neuroplasticity. On the other hand, PBM acts on cellular recovery and reduces inflammation^(18)^, which could make balance and auditory system rehabilitation more favorable.

Therefore, the objective of the present study was to analyze the effects of combining PBM and VR in adults with VS and tinnitus compared to a VR and placebo group for PBM regarding the reduction of tinnitus discomfort and improvement of vestibular function, postural balance, and dynamic visual acuity through a randomized, triple-blind clinical trial.

## METHOD

### Study design

This is a randomized, triple-blind, placebo-controlled, longitudinal clinical trial. The clinical trial was conducted in accordance with the Consolidated Standards of Reporting Trials (CONSORT) guidelines. This study was approved by the Human Research Ethics Committee (*CEP*) of the Federal University of Paraíba (UFPB) (protocol no. 5,681,217). This clinical trial was conducted between October 2022 and June 2023, at a Unified Health System (*Sistema Único de Saúde – SUS*) audiology outpatient clinic and at a private clinic in the city of Maceió, Alagoas, Brazil.

### Participants

The sample consisted of volunteers with vestibular symptoms and chronic sensorineural tinnitus referred by preceptors of an otolaryngology residency program in Maceió, Alagoas, as well as spontaneous requests received through disseminating flyers on social media. These volunteers underwent a prior evaluation with an otolaryngologist at a *SUS* audiology outpatient clinic.

Volunteers had to be over 18 years of age to be included, with constant and chronic VSs (vertigo, dizziness, and vestibulovisual symptoms) and tinnitus^(3,10)^ for more than 6 months. Pure-tone hearing thresholds had to be symmetrical in both ears, with normal hearing or hearing loss up to mild, according to the Lloyd and Kaplan classification^(22)^, or with a mild descending audiometric configuration in the high frequencies^(22)^.

The presence of signs of vestibular hypofunction in one or more semicircular canals was diagnosed by one or more of the following tests that assess the vestibulo-ocular reflex (VOR) at different frequencies^(4)^: the Caloric Test, which assesses the lateral canals at low frequencies; the Head Shake Test and Instrumental Vibration Test, which assess the VOR at intermediate frequencies; and the Video Head Impulse Test (VHIT), which assesses the VOR at high frequencies, enabling identification of deficits in specific canals. The absence of positional signs was confirmed by the Dix-Hallpike maneuvers, the Roll Test, and the Side-Lying Test, used to rule out benign paroxysmal positional vertigo (BPPV). Finally, the patient could not be taking any prescribed medication intended to improve the investigated symptoms.

The following exclusion criteria were established to ensure sample homogeneity: Central nervous system diseases; Ménière’s disease, due to the fluctuating nature of its symptoms; patients who had previously undergone vestibular rehabilitation; those taking medications to treat psychiatric disorders and sleep disorders; those undergoing hearing adaptation using a hearing aid; tinnitus modulated by muscle movements; and those over 60 years of age, as imbalance in older adults in addition to peripheral damage is multifactorial, including multisensory deficits, which can interfere with diagnostic workup; and finally, conductive and mixed hearing loss^(22)^.

### Sample size

We considered an effect size of 0.8, a margin of error of 5%, and a 95% confidence level to calculate the sample size. We estimated 26 volunteers. The calculation was performed using the online tool Sample Size Calculator for Comparing Two Means, available on the Cleveland Clinic site^(23)^.

After signing the Informed Consent Form (ICF), the volunteers underwent initial assessments (questionnaires, visual analog scales, posturography, and dynamic visual acuity tests). If audiometry and vestibular testing were not available, the volunteer was referred to the collection sites for these tests, free of charge.

### Randomization

Volunteers were randomly assigned in a 1:1 ratio. Subjects were allocated to groups using the EXCEL program using stratified randomization. The Control Group (CG) comprised volunteers who received VR and placebo PBM therapeutic intervention, and the Research Group (RG) received the same therapeutic intervention combined with active PBM. Randomization was performed in blocks; a new allocation was performed for every four participants included in the study by a researcher who was not involved in the participant assessment and intervention processes.

### Blinding

All clinical assessments and the VR intervention were conducted by speech-language pathologist “A,” who was blinded to treatment allocation. Neither speech-language pathologist “A” nor the volunteers knew whether a placebo or active treatment was being administered. The same PBM devices were used in both groups, and the PBM irradiation was administered by speech-language pathologist “B,” trained only for the transmeatal irradiation stage. The device in the CG was inserted into the external auditory canal in both ears, but the device was not activated; irradiation was performed in the RG by activating the devices. Furthermore, the volunteers wore opaque glasses to assist with blinding and visual protection. The statistician involved in the main analyses was also blinded to group allocation until the end of the statistical analyses. Only the researcher who performed the randomization and the speech-language pathologist “B,” who administered the PBM treatment, knew the participant allocation.

### Outcomes (assessments performed)

The volunteers underwent a pre-intervention assessment that included: Tinnitus Handicap Inventory (THI), Dizziness Handicap Inventory (DHI), Visual Analog Scale (VAS) for VS and Tinnitus, Dynamic Visual Acuity Test, and Posturography.

The instruments selected for the study were administered twice: before the intervention began and after 10 treatment sessions.

### Tinnitus Handicap Inventory (THI)

The THI is an instrument used to assess the degree of discomfort caused by tinnitus^(24)^. It was administered in an interview format, and the volunteer chose one of three possible responses to each of the twenty-five questions: “yes” (four points), “no” (zero points), or “sometimes” (two points). Each question relates to one of the domains: functional, emotional, or catastrophic. The functional domain (eleven items) relates to functional limitations in mental, social/occupational, and physical functioning; the emotional domain (nine items) relates to anger, frustration, irritability, and depression; and the catastrophic domain (five items) relates to despair, loss of control, inability to cope and escape, and fear of illness. The sum of the scores obtained could thus range from zero to 100.

### Dizziness Handicap Inventory (DHI)

The DHI is a validated scale for assessing the impact of dizziness on quality of life^(25)^. The DHI was also administered as an interview. It assesses the following aspects: emotional and functional, with nine questions each; and physical aspects, with seven questions, totaling 25 items. The permitted responses are “yes,” equivalent to four points; “sometimes,” equivalent to two points; and “no,” equivalent to zero. The score ranges from zero to 100 points, with closer to 100 points indicating greater the disadvantage caused by dizziness in the patient’s life.

### Visual Analog Scale (VAS)

The VAS is a psychometric response scale that ranges from zero to 10^(26)^. The researcher in charge asked the research subject to rate the discomfort intensity of their tinnitus and subsequently of the VSs. The closer to 10, the greater the discomfort reported^(26)^.

### Dynamic Visual Acuity (DVA) test

The Dynamic Visual Acuity test^(27)^ from the American Institute of Balance (AIB)^®^ system was used in this study. The assessment was conducted under three conditions: head stationary, horizontal head movement, and vertical head movement. The patient was positioned two meters away from the screen. The numbers were programmed to appear automatically, in different sizes and fonts. The patient was instructed to read each line of numbers aloud for three seconds. A metrometer indicated the speed at which the head should be moved. The decrease in visual acuity was calculated by comparing the smallest line correctly identified in the static condition with that identified during horizontal and vertical movements. The difference between these lines was recorded as the number of lines lost, according to the protocol (AIB).

### Posturography

Posturography is a technique used to objectively assess postural stability, based on recording the body’s center of pressure in different sensory conflict situations^(28)^. The model used in this study was Hórus^®^ from Contronic^®^. The participants’ stability limit was initially measured, defined as the maximum area in which the individual can voluntarily shift their center of pressure in the anterior, posterior, and lateral directions without losing balance. This parameter is calculated by the difference between the maximum displacements achieved in each direction, resulting in a total area expressed in square millimeters (mm^2^)^(28)^.

Furthermore, the equipment provides the confidence area, which represents the statistically estimated region where most postural oscillation occurs, serving as an indicator of the variability and control of the center of pressure during the task^(28)^. The participants were subsequently subjected to seven sensory conditions on the static posturography platform with dynamic tests, namely: 1- Eyes open on a stable surface; 2- Eyes closed on a stable surface; 3- Eyes open on an unstable surface; 4- Eyes closed on an unstable surface; 5- Optokinetic on the right on an unstable surface; 6- Optokinetic on the left on an unstable surface; and 7- Tunnel on an unstable surface.

### Interventions

**Figure 1:**
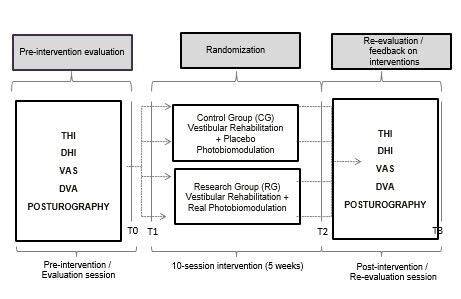
Intervention flowchart. Source: Prepared by the author

Figure 1. Demonstrates the flowchart of interventions.

VAS = Visual Analogue Scale; THI = Tinnitus Handicap Inventory; DHI = Dizziness Handicap Inventory; DVA = Dynamic Visual Acuity

### Vestibular Rehabilitation (VR)

The VR program was offered in a paired therapy format or individually when paired was not possible. Sessions were supervised by speech-language pathologist “A” and administered twice a week for five weeks. Each session lasted an average of 30 minutes. The sessions followed a standardized protocol structured by the authors and adapted from classic rehabilitation programs described in the literature, including the Cawthorne and Cooksey exercises^(29)^, the Associazione Otologi Ospedalieri Italiani (AOOI) protocol^(29)^, the Davis and O'Leary exercises^(29)^, and the protocols proposed by Herdman^(29)^ and the Federal University of São Paulo (UNIFESP)^(30)^ (Appendix A). The VR program included exercises for habituation, adaptation, gaze stabilization, balance, and gait. Stationery items such as a styrofoam ball, colored straws, post-it notes for marking fixed points, a list of pseudowords and pictures for rapid naming, and a cushion for training on an unstable surface were used during the sessions.

Volunteers were instructed to repeat one of the seven exercises proposed in the session at home two to three times a day. The selected exercise should induce dizziness or instability. Although the protocol was standardized for all participants, individual adaptations were made to effectively promote neuroplasticity^(31)^.

### Photobiomodulation (PBM)

PBM therapy was always performed after the VR session. The application was performed in a private room with the patient lying on a stretcher, using two 100mW MMO low-intensity direct current DUO laser devices (binaurally). The 808 nm (infrared) wavelength was simultaneously and bilaterally used in continuous emission in the external auditory canal, with 42 J of energy per device, totaling 84 J per session. The infrared option was selected on the device to provide continuous emission for seven minutes, according to a protocol standardized in a previous study by the authors of this study.

The Laser Duo Model 2.0 laserpuncture nozzle tip was used to better approximate the target structures, twice a week. The laserpuncture nozzle was used to bring the target tissue closer to the light beam, allowing stimulation as close to the tympanic membrane as possible. Infrared radiation was also chosen because it offers greater depth of reach to the target tissue, given that the target area will not be directly contacted.

The PBM intervention was conducted based on a protocol previously standardized by the authors in a pilot study involving 60 ears of asymptomatic adults. This study aimed to evaluate the safety of the proposed protocol by analyzing the occurrence of possible adverse effects, as well as to investigate its effectiveness in promoting measurable electrophysiological changes. Although the data from this study have not yet been published, the results obtained supported adopting this protocol in the present investigation, ensuring safe and potentially effective application parameters.

### Statistical methods

Statistical analysis was performed using the Statistical Package for the Social Sciences (SPSS) version 19.0 (SPSS Inc., Chicago, IL). The Kolmogorov-Smirnov and Shapiro-Wilk tests were used to assess the normality of the data distribution (p > 0.05), which demonstrated a normal distribution. Statistical significance was tested using global linear models in the SPSS toolbox for repeated measures and pairwise comparisons to identify differences using the Bonferroni test. Furthermore, a Student’s t-test was used to compare means, and median analysis was performed using the Mann-Whitney test. Clinical significance was assessed using the minimum detectable change (MDC%). The MDC, with 95% confidence interval, is calculated from the standard error of measurement (SEM) to indicate a true change in the CG and RG.

## RESULTS

**Figure 2:**
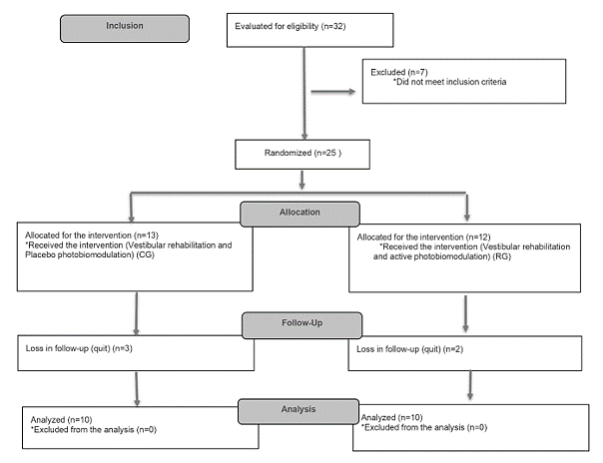
Participant flowchart, according to the Consolidated Standards of Reporting Trials (CONSORT 2010)

Source: Prepared by the author

A total of 32 volunteers expressed interest in the study after the initial screening (Figure 2). However, seven of them could not be included because VSs, tinnitus, and associated factors did not meet the research criteria. Therefore, 25 patients were randomized and included in the study. In turn, five patients dropped out during treatment. Finally, a total of 20 subjects were included in the final analysis: 10 from the control group and 10 from the research group.

Regarding audiometric thresholds, five volunteers with normal hearing, five with mild sensorineural hearing loss, and 10 subjects with mild sloping audiometric configuration in the high frequencies were included. Both the CG and the RG consisted of nine women and one man. Table 1 presents the age data of the participants. The mean age was 52.50 years (SD = 8.41) in the CG and 51.70 years (SD = 10.36) in the RG.

**Table 1:** Description and comparison between groups using the age parameter

Variable	Group	Minimum	Maximum	Mean	Standard deviation	Test statistic	p-value
Age	CG	38	60	52.50	8.410	48.500	0.912^1^
	RG	30	60	51.70	10.361		

1 Mann-Whitney test;

Legend: CG = Control Group; RG = Research Group

Source: Research data, 2023

VS decreased in both groups after the proposed therapeutic intervention process. The subjective intensity of VS measured by VAS, the physical, emotional, functional, and total DHI scores, and the Composite Balance Index showed improvements in both groups, with no statistical difference. The Sensory Analysis of Vestibular Function parameter showed a significant difference between pre- and post-therapy (p=0.003) only in the RG.

The mean stability limit area of ​​the CG increased by 1688.19 mm^2^ after the 10 VR sessions, while there was an increase of 3516.61 mm^2^ in the RG. However, no statistically significant difference was found. Table 2 shows the comparison between the groups of the aforementioned outcome parameters.

**Table 2:** Descriptive and comparative data of the groups in the pre- and post-therapy moments

**Variable**			**Minimum**	**Maximum**	**Mean**	**Standard deviation**	**Test statistic**	**p-value**
**VAS Tinnitus**	Control Group (CG)	Pre-therapy	5	10	7.60	1.955	1.435^1^	0.170
		Post-therapy	4	9	6.50	1.434		
	Research Group (RG)	Pre-therapy	4	10	8.30	2.359	15.000^2^	0.007*
		Post-therapy	1	10	4.10	3.247		
**VAS Dizziness**	Control Group (CG)	Pre-therapy	2	9	5.9	2.234	3.556^1^	0.003^*^
		Post-therapy	2	5	3.10	1.101		
	Research Group (RG)	Pre-therapy	5	10	7.70	1.829	6.394^1^	0.000^*^
		Post-therapy	0	6	2.30	1.947		
**Functional THI**	Control Group (CG)	Pre-therapy	8	44	28.40	11.423	0.491^1^	0.629
		Post-therapy	8	38	26.00	10.414		
	Research Group (RG)	Pre-therapy	20	44	32.00	9.238	5.268^1^	0.000^*^
		Post-therapy	0	28	11.40	8.222		
**Emotional THI**	Control Group (CG)	Pre-therapy	6	34	22.40	10.362	0.564^1^	0.580
		Post-therapy	4	34	19.80	10.261		
	Research Group (RG)	Pre-therapy	8	36	22.00	11.700	10.500^2^	0.002*
		Post-therapy	0	32	7.20	9.670		
**Catastrophic THI**	Control Group (CG)	Pre-therapy	8	18	13.20	3.910	1.489^1^	0.157
		Post-therapy	0	20	9.80	6.070		
	Research Group (RG)	Pre-therapy	4	20	13.60	5.948	3.526^1^	0.003*
		Post-therapy	0	14	5.40	4.326		
**Total THI**	Control Group (CG)	Pre-therapy	30	96	64.00	23.438	37.000^2^	0.353
		Post-therapy	24	88	55.80	24.284		
	Research Group (RG)	Pre-therapy	40	100	67.60	25.920	4.094^2^	0.001*
		Post-therapy	0	74	24.60	20.764		
**Physical DHI**	Control Group (CG)	Pre-therapy	12	28	20.80	5.750	4.850^1^	0.000*
		Post-therapy	4	18	9.60	4.502		
	Research Group (RG)	Pre-therapy	16	36	23.80	5.692	7.576^1^	0.000*
		Post-therapy	2	14	6.80	4.237		
**Emotional DHI**	Control Group (CG)	Pre-therapy	8	32	20.00	7.542	4.359^1^	0.001^*^
		Post-therapy	4	16	8.20	4.050		
	Research Group (RG)	Pre-therapy	12	32	21.80	7.208	5.967^1^	0.000*
		Post-therapy	0	16	5.80	4.467		
**Functional DHI**	Control Group (CG)	Pre-therapy	12	28	21.40	5.892	4.836^1^	0.000^*^
		Post-therapy	6	18	10.60	3.893		
	Research Group (RG)	Pre-therapy	16	30	23.00	5.185	9.000^2^	0.001^*^
		Post-therapy	4	26	8.60	7.427		
**Total DHI**	Control Group (CG)	Pre-therapy	40	82	61.60	15.284	5.878^1^	0.000^*^
		Post-therapy	20	48	28.20	9.449		
	Research Group (RG)	Pre-therapy	44	92	68.60	14.909	1.500^2^	0.000^*^
		Post-therapy	8	46	21.20	13.037		
**Limit of Stability**	Control Group (CG)	Pre-therapy	8710.4	16321.1	12140.060	2633.621	-1.354^1^	0.193
		Post-therapy	9879.6	17385.1	13828.250	2935.434		
	Research Group (RG)	Pre-therapy	7195.7	19269.3	12368.880	3619.764	-1.798^1^	0.091
		Post-therapy	10289.5	27289.1	15885.490	5013.693		
**Sensory Analysis of Vestibular Function**	Control Group (CG)	Pre-therapy	40.0	97.6	84.150	16.746	31.000^2^	0.165
		Post-therapy	89.2	98.4	93.370	3.315		
	Research Group (RG)	Pre-therapy	45.5	95.5	81.540	15.956	12.000^2^	**0.003** ^*^
		Post-therapy	90.1	97.0	94.710	2.2708		
**Composite Balance Index**	Control Group (CG)	Pre-therapy	50.6	95.7	85.270	14.1144	16.000^2^	0.009*
		Post-therapy	81.4	97.5	93.910	4.7864		
	Research Group (RG)	Pre-therapy	40	100	67.60	25.920	19.000^2^	0.019*
		Post-therapy	81.4	97.5	93.910	4.7864		
**DVA Horizontal score**	Control Group (CG)	Pre-therapy	72	90	80.00	5.812	-3.632^1^	0.003*
		Post-therapy	80	100	89.20	5.514		
	Research Group (RG)	Pre-therapy	42	96	71.60	17.859	12.000^2^	0.002*
		Post-therapy	80	100	92.20	6.356		
**DVA Vertical score**	Control Group (CG)	Pre-therapy	72	92	81.00	6.055	-3.632^1^	0.002*
		Post-therapy	84	100	92.20	6.563		
	Research Group (RG)	Pre-therapy	48	88	69.00	14.275	-4.100^1^	0.001*
		Post-therapy	72	100	90.60	8.592		

1 Independent samples t-test;

2 Mann-Whitney test;

* Significant data

Legend: CG = Control Group; RG = Research Group; VAS = Visual Analog Scale; THI = Tinnitus Handicap Inventory; DHI = Dizziness Handicap Inventory; DVA = Dynamic Visual Acuity

Source: Research data, 2023

Table 3 shows that Dynamic Visual Acuity in vertical scores was different between the groups (p=0.031) at the pre-intervention time point, demonstrating a greater VOR deficit in vertical movements in the RG before treatment. Nevertheless, the difference did not persist post-intervention. Dynamic Visual Acuity in the horizontal and vertical scores showed improvement in both groups, with an average of 11% in the CG and 21.6% in the RG average after therapy, with the significance index being more evident in the intervention group (RG).

**Table 3:** Descriptive and comparative data between pre- and post-therapy groups

**Variable**			**Minimum**	**Maximum**	**Mean**	**Standard deviation**	**Test statistic**	**p-value**
**VAS Tinnitus**	Pre-therapy	CG	5	10	7.60	1.955	40.000^1^	0.481
		RG	4	10	8.30	2.359		
	Post-therapy	CG	4	9	6.50	1.434	0.213^2^	0.046^*^
		RG	1	10	4.10	3.247		
**VAS Dizziness**	Pre-therapy	CG	2	9	5.9	2.234	-1.972^2^	0.065
		RG	5	10	7.70	1.829		
	Post-therapy	CG	2	5	3.10	1.101	1.131^1^	0.277
		RG	0	6	2.30	1.947		
**Functional THI**	Pre-therapy	CG	8	44	28.40	11.423	-0.775^2^	0.448
		RG	20	44	32.00	9.238		
	Post-therapy	CG	8	38	26.00	10.414	3.480^2^	0.003^*^
		RG	0	28	11.40	8.222		
**Emotional THI**	Pre-therapy	CG	6	34	22.40	10.362	48.000^1^	0.912
		RG	8	36	22.00	11.700		
	Post-therapy	CG	4	34	19.80	10.261	12.000^1^	0.002^*^
		RG	0	32	7.20	9.670		
**Catastrophic THI**	Pre-therapy	CG	8	18	13.20	3.910	-0.178^2^	0.861
		RG	4	20	13.60	5.948		
	Post-therapy	CG	0	20	9.80	6.070	1.867^2^	0.080
		RG	0	14	5.40	4.326		
**Total THI**	Pre-therapy	CG	30	96	64.00	23.438	-0.326^2^	0.748
		RG	40	100	67.60	25.920		
	Post-therapy	CG	24	88	55.80	24.284	-4.326	0.000^*^
		RG	0	74	24.60	20.764		
**Physical DHI**	Pre-therapy	CG	12	28	20.80	5.750	-0.172^2^	0.256
		RG	16	36	23.80	5.692		
	Post-therapy	CG	4	18	9.60	4.502	1.432^2^	0.169
		RG	2	14	6.80	4.237		
**Emotional DHI**	Pre-therapy	CG	8	32	20.00	7.542	-0.546^2^	0.592
		RG	12	32	21.80	7.208		
	Post-therapy	CG	4	16	8.20	4.050	30.500^1^	0.143
		RG	0	16	5.80	4.467		
**Functional DHI**	Pre-therapy	CG	12	28	21.40	5.892	-0.645^2^	0.527
		RG	16	30	23.00	5.185		
	Post-therapy	CG	6	18	10.60	3.893	25.500^1^	0.063
		RG	4	26	8.60	7.427		
**Total DHI**	Pre-therapy	CG	40	82	61.60	15.284	-1.037^2^	0.314
		RG	44	92	68.60	14.909		
	Post-therapy	CG	20	48	28.20	9.449	3.480^2^	0.159
		RG	8	46	21.20	13.037		
**Limit of Stability**	Pre-therapy	CG	8710.4	16321.1	12140.060	2633.6216	-0.162^2^	0.874
		RG	7195.7	19269.3	12368.880	3619.7642		
	Post-therapy	CG	9879.6	17385.1	13828.250	2935.4343	39.000^1^	0.436
		RG	10289.5	27289.1	15885.490	5013.6937		
**Sensory Analysis of Vestibular Function**	Pre-therapy	CG	40.0	97.6	84.150	16.746	43.000^1^	0.631
		RG	45.5	95.5	81.540	15.956		
	Post-therapy	CG	89.2	98.4	93.370	3.315	-1.055^2^	0.307
		RG	90.1	97.0	94.710	2.2708		
**Composite Balance Index**	Pre-therapy	CG	50.6	95.7	85.270	14.114	46.000^1^	0.796
		RG	40	100	67.60	25.920		
	Post-therapy	CG	81.4	97.5	93.910	4.7864	46.500^1^	0.791
		RG	81.4	97.5	93.910	4.7864		
**DVA Horizontal score**	Pre-therapy	CG	72	90	80.00	5.812	40.000^1^	0.481
		RG	42	96	71.60	17.859		
	Post-therapy	CG	80	100	89.20	5.514	-1.127^2^	0.275
		RG	80	100	92.20	6.356		
**DVA Vertical score**	Pre-therapy	CG	72	92	81.00	6.055	2.447^2^	**0.031** ^*^
		RG	48	88	69.00	14.275		
	Post-therapy	CG	84	100	92.20	6.563	0.468^2^	0.646
		RG	72	100	90.60	8.592		

1 Mann-Whitney test;

2 Independent samples t-test;

* Significant data

Legend: CG = Control Group; RG = Research Group; VAS = Visual Analog Scale; THI = Tinnitus Handicap Inventory; DHI = Dizziness Handicap Inventory; DVA = Dynamic Visual Acuity

Source: Research data, 2023

In contrast, only the RG demonstrated a statistically significant improvement in the pre- and post-therapy moments in relation to the tinnitus evaluation parameters, as well as in the comparison between the groups in the post-therapy moment (Table 3); VAS Tinnitus (p=0.007), Functional THI (p=0.000), Emotional THI (p=0.002), Total THI (p=0.003). There was no significant difference in the Catastrophic THI (p=0.080).

## DISCUSSION

The mean age in the present study was 52.50 years in the control group (CG) and 51.70 years in the research group (RG). The most common hearing loss was mild, sloping sensorineural hearing loss in the high frequencies. This finding is consistent with the audiometric patterns described in the literature for presbycusis, characterized by progressive, bilateral, and symmetrical hearing loss, with greater impairment in the high frequencies^(10)^.

In addition to hearing changes, vestibular dysfunctions become more prominent with advancing age, suggesting a possible association between degeneration of the auditory and vestibular systems throughout the aging process^(32)^. Studies indicate that these changes may be related to common pathophysiological mechanisms, such as neuronal degeneration in the vestibulocochlear nerve and changes in the hydrodynamics of inner ear fluids, which affect both the cochlea and the semicircular canals^(33)^. 

Therefore, considering that both tinnitus and vestibular symptoms are more prevalent in older individuals^(14)^, it is possible that the pathophysiology of aging played a relevant role in the research findings, being characterized by changes in calcium homeostasis and loss of peripheral neural function^(34,35)^.

A study^(36)^ in rodents demonstrated that this reduction in calcium-binding proteins is associated with an increase in the electrophysiological threshold of Brainstem Auditory Evoked Potential (BAEP) and a reduction in the amplitude of distortion product otoacoustic emissions in older rodents.

Another systematic review study^(37)^ identified that one of the molecular mechanisms triggered after PBM is increased ATP production, which induces an intracellular calcium influx and enables regulating toxic intracellular calcium levels. An *in vitro* and *in vivo* study^(20)^ of cochlear cells demonstrated positive effects of PBM, such as reduced oxidative stress levels, histological recovery of cells after treatment with aminoglycosides, and improved recovery of electrophysiological thresholds after induced acoustic trauma. These findings reinforce the need for further investigation to clarify whether the response to PBM can be modulated by age factors and whether this therapy offers greater benefits in populations with aging-related metabolic impairments.

A systematic review with meta-analysis^(19)^ suggests that patients with tinnitus resulting from acoustic trauma or auditory nerve degeneration may benefit more from PBM compared to other possible tinnitus etiologies. This finding may explain the positive results observed in the present study.

The present study also demonstrated differences between the groups in tinnitus parameters (VAS and THI), which corroborates research within the otoneurological clinic that demonstrates positive outcomes from the use of PBM in patients with tinnitus^(38-42)^.

Mollasadeghi et al.^(37)^ investigated patients with bilateral sensorineural hearing loss who underwent 20 sessions of photobiomodulation (PBM) with different parameters (650 nm, 5 mW, mastoid stimulation). Similarly, another study^(39)^ used an infrared wavelength (830 nm) with a power of 100 mW, similar to the protocol adopted in the present study, but with an irradiated energy of 120 J per session. This study found improvements in tinnitus intensity and duration, but no statistical differences in VAS and THI scores. Furthermore, it was observed that participants in the group who did not experience improvement had significantly worse hearing thresholds than those who obtained benefits.

A recent study^(40)^ reinforces that the positive effect of PBM depends on the different application settings. The aforementioned clinical trial^(40)^ indicated that the group with the best THI outcomes used a transmeatal red laser prototype (660 nm, 100 mW, 180 J per session) with bilateral application, presenting superior results compared to participants who received a lower energy dose from the same equipment (72 J per session). It is noteworthy that wavelength influences tissue penetration, with 660 nm tending to reach more superficial layers, while 808 nm presents greater absorption depth^(18-20,37)^. This difference may explain the positive effects observed in the present study, which adopted an 84 J per session infrared configuration, with a tip closer to the tympanic membrane.

Another study^(41)^ analyzed the impact of PBM with an infrared wavelength (830 nm, 67 mW, 80.4 J/cm^2^) in 12 sessions spread over four weeks. The results showed statistically significant differences in VAS and THI when compared to the control group. A study^(42)^ which used equipment with a power of 5 mW, wavelength of 650 mm for 20 minutes (6 J per session) in divers with normal hearing and the presence of tinnitus presented significant results after 40 and 60 sessions; however, the authors used a non-validated scale as a criterion to evaluate the outcome.

Despite the positive findings previously reported, some studies have shown divergent results, with no significant distinction between the intervention group and the placebo group^(43-46)^. Among these, only one study^(43)^ used a laser with a power of 100 mW, a wavelength of 606 nm, and applied transmeatal with a dose of 4 J per session, without demonstrating statistically significant differences between the groups.

Furthermore, other studies^(44-46)^ investigated transmeatal PBM using devices with a power lower than 7 mW and wavelengths in the red range, but also did not observe positive effects in the treatment of idiopathic tinnitus. These findings suggest that factors such as power and application parameters may influence therapeutic outcomes.

Regarding the VS findings, the present study demonstrated the effectiveness of the VR program developed by the authors, as evidenced by the significant reduction in total DHI scores in the post-treatment phases in both groups. These findings corroborate the literature^(2,3,8,9)^, which points to VR as an effective approach in vestibular hypofunction, with moderate to strong evidence, reinforcing the results obtained in this study. However, the association with PBM demonstrated additional potential in improving variables such as sensory analysis of vestibular function and the stability limit, which suggests that photobiomodulation may act synergistically, promoting neuroplastic and restorative effects in the vestibular system.

A statistically significant improvement was observed regarding posturography parameters in the Sensory Analysis of Vestibular Function in the RG. Although there was an increase in the mean post-intervention Limit of Stability (LoS) in both groups, this was also greater in the RG. LoS is the individual’s ability to voluntarily shift their center of mass with precision and speed, without altering their support base^(28,46)^. This parameter is proportionally related to the risk of falls. A study^(46)^ demonstrated that an increase in LoS is correlated with improvement in the sensory analysis of vestibular function, which corroborates our findings. We found no differences in the Composite Balance Index.

In turn, both the RG and the CG showed statistically significant improvements between pre- and post-therapy regarding dynamic visual acuity. However, the group that underwent true PBM showed a significantly greater increase in the mean horizontal and vertical scores compared to the CG. Visual acuity measurement during head movement has been used to assess the functionality and impact of vestibular hypofunction. Changes in visual acuity impact patients’ daily activities, such as driving, reading, and watching television^(27)^. The test has been reported in the literature as reliable in terms of sensitivity and specificity, being able to distinguish normal individuals from patients with vestibular loss^(27)^.

No clinical trials with bilateral transmeatal PBM with infrared wavelengths were found in patients with tinnitus, nor in PBM for patients with vestibular alterations, regardless of the irradiation parameter.

To the best of our knowledge, this study is the first prospective randomized controlled clinical trial demonstrating the beneficial effects of PBM combined with VR in individuals with VSs and tinnitus. The limitation of this study is that the small number of volunteers may not be sufficient to draw firm conclusions about the clinical effects. Although the underlying disease was not considered in the study analysis, clinical signs were delimited in the inclusion and exclusion criteria, which enabled groups with homogeneous characteristics and symptoms.

We found no improvement in tinnitus symptom parameters in the group that received placebo PBM, therefore VR was unable to intervene in this symptom. We also found no clinical trials using VR for tinnitus treatment. Further longitudinal and prospective studies are needed to evaluate the long-term results of the effects of PBM combined with VR on VSs, as well as PBM combined with scientifically proven interventions for tinnitus, using a larger sample size and long-term follow-up.

## CONCLUSION

The results of this study demonstrate that photobiomodulation (PBM) associated with Vestibular Rehabilitation (VR) presented significant effects in reducing tinnitus discomfort, as assessed by VAS and THI, as well as in the Sensory Analysis of Vestibular Function, analyzed by posturography, when compared to the placebo group. There was also an improvement in the Dynamic Visual Acuity and Limit of Stability parameters in relation to the Control Group, but without statistical effect. No differences were observed between the groups in relation to VAS Dizziness, DHI and Composite Balance Index, nor were any positive effects of VR found for the tinnitus symptom.

## Appendix A Vestibular rehabilitation protocol

**Table:**

**1st week, sessions 1 and 2**
1. Look up and down, aiming at two fixed points, slowly and then quickly (standing) (2 minutes).
2. Look right and left, aiming at two fixed points, slowly and then quickly (standing) (2 minutes).
3. Move a multicolored straw closer and further away (visual convergence), looking at it (standing) (slowly and then quickly) (2 minutes).
4. Toss a ball from one hand to the other, following it with your eyes (30 times).
5. Place an object on the floor. Pick it up and raise it above your head, then place it back down (looking at the object the entire time) (3 sets of 10 repetitions).
6. Sit, then stand and turn to one side, sit again, stand again, and now turn to the other side (15 times).
7. Turn your head 45 degrees from side to side, without stopping, keeping your gaze focused on an “X” on a card in front of you, for one to two minutes, slowly the first time and quickly the second (2 minutes).
**2nd week, sessions 3 and 4**
1. While sitting, turn your head to the right, look up and down, and back, following these eye movements, while moving your head at the same time. (10 repetitions). While sitting, turn your head to the left, look up and down, and back, following these eye movements, while moving your head at the same time (10 repetitions) (Perform two sets).
2. Turn your head 45 degrees from side to side, pausing briefly with your head centered, keeping your gaze focused on an “X” on a card in front of you, for one to two minutes, starting slowly and then quickly (standing) (2 minutes).
3. Repeat the previous exercise, moving vertically (standing) (2 minutes).
4. Move your head in flexion and extension with your eyes open, in a vertical plane, as if nodding “yes,” keeping your gaze fixed (standing) (slowly and then quickly looking at a fixed point on the wall) (3 minutes). If you feel dizzy, stop for 10 seconds and start again, until you reach the 3-minute mark. Move your head to the right and left with your eyes open, head movements in a horizontal plane, saying “no,” keeping your gaze fixed (standing) (slowly and then quickly). If you feel dizzy, stop for 10 seconds and start again, until you reach the 3-minute mark.
5. Take a step and rotate your neck to the right and left, walking forward and backward (3 minutes).
6. Stand with your feet as far apart as possible, aiming at a target in front of you. Progressively narrow the base of support by successively placing your feet together, one foot partially in front of the other, with your arms open, then alongside your body and finally crossed over your chest (15 times).
**3rd week, sessions 5 and 6**
1. Stand with your feet as far apart as possible, head bent 30 degrees forward, arms open, then at your sides, and finally crossed over your chest (15 repetitions).
2. Stand with your feet as far apart as possible, head bent 30 degrees back, arms open, then at your sides, and finally crossed over your chest (15 times).
3. Move your head in flexion and extension with your eyes open (walking forward and backward (when possible) (quickly looking at a fixed point on the wall) (3 minutes). If you feel dizzy, stop for 10 seconds and start again, until you reach 3 minutes of exercise.
4. Move your head to the right and left with your eyes open (walking forward and backward (when possible) (quickly looking at a fixed point on the wall). If you feel dizzy, stop for 10 seconds and start again, until you reach 3 minutes of exercise.
5. Walk with one foot in front of the other with your eyes open, facing forward (this exercise can be performed with hand support, if necessary) (3 minutes).
6. Walk and rotate your neck to the right and left, walking forward and backward (when possible), without pausing between steps (3 minutes).
7. Move a card and your head in opposite directions, in the horizontal plane, without stopping, focusing on a list of words (2 minutes).
**4th week, sessions 7 and 8**
1. Move a card and your head in opposite directions, without stopping, focusing on a list of pseudowords (2 minutes), repeat vertically (2 minutes).
2. March in place on a pillow, looking straight ahead, 10 seconds with your eyes closed and 10 seconds with your eyes open (10 times) (exercise can be performed with hand support).
3. On a pillow, move your head, bending it from side to side, looking at a fixed point, 10 seconds with your eyes open and 10 seconds with your eyes closed (exercise can be performed with hand support).
4. Stand in front of the other person, with your eyes open, on the pillow, 1 minute in each standing position (exercise with therapist support).
5. Walk forward, one foot in front of the other, with your eyes open, moving your eyes to the right and left (3 minutes).
6. March (walking) and forming a figure 8, looking at a card with an X on it (2 minutes).
7. March taking five steps forward and turning 360 degrees to the right, then take another five steps (marching) and turning 360 degrees to the left (2 minutes).
**5th week, sessions 9 and 10**
1. Marching forward five steps and turning 360 degrees to the right, then taking another five steps (marching) and turning 360 degrees to the left (eyes closed) (2 minutes).
2. Marching in place on a pillow, moving the head to the right and left with eyes open, head movements in a horizontal plane, expressing a “no” while keeping the gaze fixed on a point (2 minutes).
3. Marching in place on a pillow, moving the head in a vertical plane, as if the patient were nodding “yes” while keeping the gaze fixed on a point. (2 minutes)
4. Walking with one foot in front of the other, with eyes open, forward, moving the head to the right and left (2 minutes) (this exercise can be performed with hand support).
5. Marching in a circular motion on a chair while looking at fixed points to the right and left 10 times in each direction (2 sets).
6. Walk and move your head to the right and left with your eyes open, head movements in the horizontal plane, saying “no” (2 minutes, with your eyes open) (This exercise can be performed with hand support, near a wall).
7. Walk and walk forward, moving your head, bending it from side to side, while quickly naming figures (3 minutes).

Source: Authors (2026), adapted from Cawthorne and Cooksey, AOOI, Herdman and Davis and O’Leary cited in Morozetti et al.^(29)^, UNIFESP^(30)^
