# Supplementary material for: Effects of photobiomodulation associated with vestibular rehabilitation in the treatment of patients with vestibular symptoms and tinnitus: a triple-blind randomized clinical trial
Source: Codas. 2026 Feb 9;38(1):e20230305. doi: 10.1590/2317-1782/e20230305en (PMC12978320; doi:10.1590/2317-1782/e20230305en)
Supplement: Translation

# Translation: Efeitos da fotobiomodulação associada à reabilitação vestibular no tratamento de pacientes com sintomas vestibulares e zumbido: ensaio clínico randomizado triplo-cego

**DOI:** 10.1590/2317-1782/e20230305pt

**Authors:** Thales Roges Vanderlei de Góes, Marine Raquel Diniz da Rosa, Giorvan Anderson dos Santos Alves, Rafael Nóbrega Bandeira, Sávio Bastos

**Affiliations:** 1 Departamento de Fonoaudiologia, Universidade Federal da Paraíba – UFPB - João Pessoa (PB), Brasil.

**Keywords:** Doenças Vestibulares, Equilíbrio Postural, Terapia com Luz de Baixa Intensidade, Tontura, Zumbido

## Abstract

**Objetivo:**

Analisar os efeitos da Fotobiomodulação (FBM) associada à Reabilitação Vestibular (RV) em adultos com sintomas vestibulares e zumbido, em comparação com a RV associada à FBM placebo, quanto à redução do incômodo do zumbido e à melhora da função vestibular, equilíbrio postural e acuidade visual dinâmica.

**Método:**

Ensaio clínico randomizado, triplo-cego, com 20 voluntários diagnosticados com hipofunção vestibular e zumbido crônico. Os participantes foram alocados em dois grupos: grupo pesquisa (GP), submetido à RV com FBM ativa; e grupo controle (GC), submetido à RV com FBM placebo. Cada grupo realizou dez sessões, duas vezes por semana, durante cinco semanas. As avaliações pré e pós-intervenção incluíram: Tinnitus Handicap Inventory (THI), Dizziness Handicap Inventory (DHI), Escala Visual Analógica (EVA), teste de Acuidade Visual Dinâmica (AVD) e posturografia. Os dados foram analisados pelos testes t de Student e Mann-Whitney (p<0,05).

**Resultados:**

Na comparação entre os grupos no momento pós- intervenção , O GP apresentou melhora significativa na EVA para zumbido (p=0,046), nos domínios funcional (p=0,003), emocional (p=0,002) e total (p=0,000) do THI. O parâmetro Análise Sensorial da Função Vestibular mostrou diferença significante nos momentos pré e pós- terapia (p=0,003) apenas no GP. A EVA Sintomas Vestibulares, os escores do DHI e o Índice de Equilíbrio Composto apresentaram melhora nos dois grupos, sem diferença estatística.

**Conclusão:**

A FBM associada à RV reduziu significativamente o incômodo do zumbido e apresentou um desempenho superior na Análise Sensorial da Função Vestibular . A RV isolada não demonstrou efeito na redução do zumbido.

## INTRODUÇÃO

Os sintomas vestibulares (SV) são decorrentes de um desequilíbrio no processamento das informações do aparelho vestibular, visual e somatossensorial, a disfunção pode ocorrer devido a alterações em um ou mais sistemas^(1)^. Sendo, portanto, causados por uma variedade de etiologias^(2-5)^.

Dentro da definição de sintomas vestibulares, deve-se considerar o termo vertigem referente à sensação deturpada de auto movimento ocorrendo durante ou na ausência de deslocamento cefálico^(2)^. Tontura, como uma sensação de desorientação espacial^(2)^. Além desses SV, ainda pode-se incluir os vestíbulo-visuais e os posturais^(2)^.

O sucesso das medidas farmacológicas nos SV depende de um diagnóstico preciso, tratado com o medicamento adequado, em dosagem e duração apropriadas, escolhas inadequadas podem tornar o tratamento ineficaz^(2,5)^. Outra opção terapêutica para os SV é a Reabilitação Vestibular (RV), forma fisiológica de tratamento, em que são considerados os sintomas e alterações funcionais. A RV pode ser definida como um conjunto de procedimentos terapêuticos , destinados a promover plasticidade do sistema nervoso central, por meio de exercícios de adaptação, compensação e substituição vestibular^(2,3,6,7)^. Não existe um modelo único de reabilitação, o plano terapêutico deve ser elaborado de forma personalizada para os sintomas vestibulares específicos do indivíduo^(3,7-9)^. Revisões sistemáticas com metanálise^(8,9)^ e *guidelines* ^(2,3)^ demonstram evidência moderada a forte da eficácia e segurança da RV nas hipofunções vestibulares unilateral ou bilateral.

O zumbido, por sua vez, pode ser definido como a percepção consciente de um som na ausência de um estímulo auditivo externo^(10)^. Na grande maioria dos casos é classificado como subjetivo, ou seja, apenas o indivíduo afetado consegue ouvir o som^(10)^. A prevalência de zumbido aumenta com a idade, cerca de 9,7% da população mundial de adultos jovens entre 18 e 44 anos percebem o zumbido, esse índice aumenta para 23,6% em pessoas acima de 65 anos. A prevalência combinada de zumbido grave, ou seja, que relata incômodo, entre adultos é estimada em 2,3%^(11)^. Apesar de sua fisiopatologia ser multifatorial e controversa, o zumbido poderia ser resultado de uma atividade neural anormal em qualquer nível do sistema auditivo^(12)^.

O zumbido e os SV podem ocorrer de forma simultânea ou independentemente^(13)^. A associação dos sintomas é muito comum, no processo de envelhecimento^(14)^, na migrânea vestibular^(15)^, Ménière^(16)^ e transtornos emocionais^(17)^. Ambos trazem prejuízos à qualidade de vida do indivíduo, o que pode limitar as atividades diárias^(14-17)^.

Assim como a origem do zumbido, as terapêuticas são diversificadas^(18)^. Quando existe uma causa identificável, ela deve ser tratada. O controle da causa pode não ser suficiente para reduzir ou eliminar o zumbido^(13)^. Com graus variáveis de sucesso, existem várias possibilidades terapêuticas, tais como, Aconselhamento Auditivo, Terapia de Retreinamento (TRT), Mascaramento Sonoro, utilização de Prótese Auditiva, Terapia Comportamental, Mindfulness, Terapia Manual e a Fotobiomodulação (FBM)^(18)^.

A terapia por FBM é uma forma de terapia de luz que utiliza formas não ionizantes, como os *LASERS* e Diodos Emissores de Luz (LEDs) que atuam como facilitadores da microcirculação sanguínea, por meio da inibição neural simpática, o que permite um aumento na proliferação celular, tendo como consequência, uma maior facilidade na síntese de trifosfato de adenosina (ATP) nas mitocôndrias^(18,19)^. Esse processo possibilita acelerar o reparo e diminuir o dano das células e tecidos irradiados^(20)^.

Os desfechos clínicos dos efeitos da FBM no zumbido ainda continuam controversos. Revisões sistemáticas^(18,19)^ analisaram estudos com efeitos positivos na percepção e incômodo do sintoma, entretanto outros não apresentaram distinção do grupo placebo. Essa discrepância pode ser causada por divergências nos métodos de irradiação utilizados, ou ainda por diferenças nas amostras dos pacientes.

Quanto ao uso da FBM nos SV, em pesquisa com modelo animal^(21)^, uma dose de gentamicina foi injetada em camundongos (macho, doze semanas) para induzir vestibulopatia. A FBM foi realizada em sete dias consecutivos. O tratamento normalizou o vestíbulo (mensuradas a partir de um teste de função vestibular que avaliou o reflexo vestíbulo-ocular) e retorno ao quase normal da histologia da cúpula, enquanto no controle, o vestíbulo permaneceu comprometido. O que demonstra que o tratamento, além de ser não invasivo e sem efeitos adversos relatados, pode ser um recurso promissor para alterações vestibulares em humanos.

Nessa perspectiva, a RV e a FBM poderiam apresentar-se como terapias complementares para os SV e zumbido. A coexistência desses sintomas pode ser atribuída a mecanismos neurofisiológicos comuns, nos quais a disfunção vestibular e a atividade neural anormal no sistema auditivo se inter-relacionam, exacerbando a experiência do paciente^(13)^. A RV, ao focar na reabilitação funcional^(2)^, visa restaurar a estabilidade postural e a funcionalidade vestibular por meio de exercícios específicos que promovem a neuroplasticidade. Por outro lado, a FBM atua na recuperação celular e na redução da inflamação^(18)^, o que poderia tornar a reabilitação do equilíbrio e do sistema auditivo mais propícia. Portanto, o objetivo do presente estudo foi analisar, em adultos com SV e zumbido, os efeitos da associação entre a FBM e a RV, em comparação à um grupo de RV e placebo para à FMB, quanto à redução do incômodo do zumbido e à melhora da função vestibular, do equilíbrio postural e da acuidade visual dinâmica, por meio de um ensaio clínico randomizado triplo-cego.

## MÉTODO

### Desenho do estudo

Trata-se de um ensaio clínico randomizado triplo-cego, controlado por placebo, de corte longitudinal. O ensaio clínico foi conduzido em conformidade com as diretrizes do *Consolidated Standards of Reporting Trials* **-**CONSORT. Este estudo recebeu aprovação do Comitê de Ética em Pesquisa (CEP) em Seres Humanos da Universidade Federal da Paraíba (UFPB), (protocolo nº 5.681.217). Este ensaio clínico foi realizado entre outubro de 2022 e Junho de 2023, em um ambulatório de audiologia do SUS e em uma clínica particular , na cidade de Maceió, Alagoas.

### Participantes

A amostra foi constituída por voluntários com sintomas vestibulares e zumbido neurossensorial crônico encaminhados por preceptores de uma residência médica, em otorrinolaringologia, da cidade de Maceió, Alagoas, além de demanda espontânea que ocorreu por meio da divulgação de *folders* em redes sociais. Estes últimos realizaram avaliação prévia com a otorrinolaringologista de um ambulatório de audiologia do SUS.

Para serem incluídos, os voluntários deveriam ter idade superior à 18 anos, com presença de SV (vertigem, tontura e sintomas vestíbulo-visuais) e zumbido de forma constante e crônica^(3,10)^, ou seja, por mais de 6 meses. Os limiares auditivos tonais deveriam ser simétricos em ambas as orelhas, com audição normal ou presença de perda auditiva, até o grau leve, segundo à classificação de Lloyd e Kaplan^(22)^, ou ainda, com configuração audiométrica descendente leve, nas frequências agudas^(22)^. Presença de sinais de hipofunção vestibular em um ou mais canais semicirculares diagnosticados, por um ou mais dos seguintes testes que avaliam o reflexo vestíbulo-ocular (RVO) em diferentes frequências^(4)^: Prova Calórica que avalia os canais laterais em baixa frequência, *Head Shake Test* e Vibração Instrumentalizada que analisam o RVO em frequência intermediária e *o Video Head Impulse Test* (VHIT) que avalia o RVO em alta frequência, permitindo identificar déficits em canais específicos. A ausência de sinais posicionais foi confirmada pelas manobras de Dix-Hallpike, Roll Test e Side-Lying Test, utilizadas para excluir vertigem posicional paroxística benigna (VPPB). E por último, não estar em uso de medicação receitada com a finalidade de melhorar os sintomas investigados.

Para garantir a homogeneidade da amostra, estabeleceram-se os seguintes critérios de exclusão: Doenças do sistema nervoso central; Doença de Ménière, devido à natureza flutuante de seus sintomas, pacientes que já realizaram reabilitação vestibular prévia; em uso de fármacos para tratamento de transtornos psiquiátricos e distúrbios do sono; em processo de adaptação auditiva por meio de aparelho de amplificação sonora individual; zumbidos modulados mediante movimentos musculares; população acima de 60 anos, pois além da lesão periférica, o desequilíbrio em idosos é multifatorial, incluindo déficits multissensoriais, o que pode interferir na propedêutica. Perdas auditivas condutivas e mistas^(22)^.

### Tamanho da amostra

Para o cálculo do tamanho da amostra, considerou-se tamanho de efeito de 0.8, margem de erro de 5% e nível de confiabilidade de 95%, foi estimado 26 voluntários. O cálculo foi realizado por meio da ferramenta online *Sample Size Calculator for Comparing Two Means*, disponível no site Cleveland Clinic^(23)^.

Após assinar o Termo de Consentimento Livre e Esclarecido (TCLE), os voluntários realizaram as avaliações iniciais (questionários, escalas visuais analógicas, os exames de posturografia e acuidade visual dinâmica). Na ausência de audiometria e exame vestibular, o voluntário era encaminhado para realizar nos locais de coleta, sem custo para o sujeito.

### Randomização

Os voluntários foram alocados de forma randomizada na proporção, 1: 1. A alocação dos sujeitos nos grupos foi realizada por intermédio do programa EXCEL utilizando a randomização estratificada. Para o Grupo Controle (GC), voluntários que receberam intervenção terapêutica de RV e FBM placebo e Grupo Pesquisa (GP), com a mesma intervenção terapêutica combinada com o uso da FBM ativa.

A Randomização ocorreu em bloco, a cada quatro participantes incluídos na pesquisa, uma nova alocação era realizada, por uma pesquisadora que não participou dos processos de avaliações e intervenções dos participantes.

### Cegamento

Todas as avaliações clínicas e a intervenção de RV foram conduzidas pelo fonoaudiólogo “A” cego para a alocação do tratamento. Nem o fonoaudiólogo “A” e nem os voluntários sabiam se estava sendo administrado um placebo ou um tratamento ativo. Os mesmos dispositivos de FBM foram utilizados nos dois grupos e a irradiação da FBM foi administrada pelo fonoaudiólogo “B” treinado apenas para etapa de irradiação transmeatal. No GC, o equipamento era introduzido no meato acústico externo, em ambas as orelhas, porém, o equipamento não era acionado, no GP a irradiação era realizada pelo acionamento dos dispositivos. Além disso, os voluntários usaram óculos opacos que auxiliaram no cegamento e na proteção visual. O estatístico envolvido nas principais análises também desconhecia a alocação dos grupos até o final das análises estatísticas. Apenas a pesquisadora que realizou a randomização e o fonoaudiólogo “B” que realizou o tratamento de FBM sabiam da alocação dos participantes.

### Desfechos (avaliações realizadas)

Os voluntários passaram por avaliação pré-intervenção que constaram de: *Tinnitus Handicap Inventory* (THI), *Dizziness Handicap Inventory* (DHI), Escala Visual Analógica (EVA) para os SV e Zumbido, Teste de Acuidade Visual Dinâmica e Posturografia.

Os instrumentos selecionados para a pesquisa foram aplicados em dois momentos, antes do início da intervenção e após dez sessões de atendimento.

### *Tinnitus Handicap Inventory* (THI)

O THI é um instrumento para verificar o grau de incômodo do zumbido^(24)^. Foi aplicado em forma de entrevista e o voluntário escolhia, umas das três possibilidades de resposta a cada uma das vinte e cinco questões: ‘‘sim’’ (quatro pontos), ‘‘não’’ (zero ponto) ou ‘‘às vezes’’ (dois pontos). Cada pergunta se relaciona com um dos domínios: funcional, emocional ou catastrófico. O domínio funcional (onze itens) está relacionado às limitações de função no funcionamento mental, social/ocupacional e físico; o domínio Emocional (nove itens), à raiva, frustração, irritabilidade, depressão; o domínio Catastrófico (cinco itens), ao desespero, perda de controle, incapacidade de lidar e escapar, medo de doença. A soma das pontuações obtidas poderia assim variar de zero a cem.

### *Dizziness Handicap Inventory* (DHI)

O DHI é uma escala validada a fim de avaliar o impacto da tontura na qualidade de vida^(25)^. O DHI também foi aplicado em forma de entrevista. Avalia os seguintes aspectos: emocional e funcional, com nove questões cada e o aspecto físico, com sete questões, no total de vinte e cinco quesitos. As respostas permitidas são "sim", equivalente a quatro pontos, "às vezes", equivalente a dois pontos e "não", equivalente a zero. A pontuação varia de zero a cem pontos, sendo que quanto mais próximo de cem, maior será a desvantagem causada pela tontura na vida do paciente.

### Escala Visual Analógica (EVA)

A EVA é uma escala de resposta psicométrica que vai de zero a dez^(26)^. O pesquisador responsável solicitava que o sujeito de pesquisa pontuasse a intensidade do incômodo do seu zumbido e posteriormente dos SV. Quanto mais próximo do dez, maior é o incômodo referido^(26)^.

### Teste de Acuidade Visual Dinâmica (AVD)

O Teste de Acuidade Visual Dinâmica^(27)^ utilizado no presente estudo foi o do *American Institute of Balance (AIB)*®, a avaliação ocorreu em três condições, cabeça parada, movimentação cefálica horizontal e movimentação vertical. O paciente foi posicionado à dois metros de distância da tela. Os números foram programados para aparecer automaticamente, em diferentes tamanhos e fontes, o paciente foi instruído a ler em voz alta, cada linha de números durante três segundos. Um metrômero indicava a velocidade em que a cabeça deveria ser movimentada. O cálculo do decréscimo da acuidade visual foi realizado comparando-se a menor linha corretamente identificada na condição estática com aquela identificada durante os movimentos horizontal e vertical. A diferença entre essas linhas foi registrada em termos do número de linhas perdidas, conforme o protocolo do (AIB).

### Posturografia

A Posturografia é uma técnica utilizada para avaliação objetiva da estabilidade postural, com base no registro do centro de pressão corporal em diferentes situações de conflito sensorial^(28)^. O modelo utilizado na presente pesquisa foi o Hórus®, da marca Contronic®. Inicialmente, foi mensurado o limite de estabilidade dos participantes, definido como a área máxima em que o indivíduo consegue deslocar voluntariamente seu centro de pressão nas direções anterior, posterior e laterais, sem perda do equilíbrio. Esse parâmetro é calculado pela diferença entre os deslocamentos máximos alcançados em cada direção, resultando em uma área total expressa em milímetros quadrados (mm^2^)^(28)^.

Além disso, o equipamento fornece a área de confiança, que representa a região estatisticamente estimada onde ocorre a maior parte da oscilação postural, servindo como um indicador da variabilidade e controle do centro de pressão durante a tarefa^(28)^.

Posteriormente os participantes foram submetidos a sete condições sensoriais na plataforma de posturografia estática com provas dinâmicas , sendo elas: 1- Olhos abertos em superfície estável, 2- Olhos fechados na superfície estável, 3- Olhos abertos na superfície instável, 4- Olhos fechados na superfície instável, 5- Optocinético à direita na superfície instável, 6- Optocinético à esquerda na superfície instável, 7- Túnel na superfície instável.

### Intervenções

A Figura 1 demonstra o fluxograma das intervenções.

**Figura 1:** Fluxograma de intervenções. Fonte: Elaborada pelo autor

EVA = Escala Visual Analógica; THI = Tinnitus Handicap Inventory; DHI = *Dizziness Handicap Inventory*; AVD = *Acuidade Visual Dinâmica*

### Reabilitação Vestibular (RV)

O programa de RV foi ofertado em formato de terapia em dupla, ou quando não possível de forma individual. As sessões foram supervisionadas pelo fonoaudiólogo “A”, administrada duas vezes por semana, durante cinco semanas. Cada sessão tinha em média, uma duração de trinta minutos. As sessões seguiram um protocolo padronizado, estruturado pelos autores e adaptado a partir de programas clássicos de reabilitação descritos na literatura, incluindo os exercícios de Cawthorne e Cooksey^(29)^, o protocolo da Associazione Otologi Ospedalieri Italiani (AOOI)^(29)^, os exercícios de Davis e O’Leary^(29)^, os protocolos propostos por Herdman^(29)^ e pela Universidade Federal de São Paulo (UNIFESP)^(30)^ (Apêndice A). O programa de RV compreendeu exercícios para habituação, adaptação, estabilização do olhar, equilíbrio e marcha. Foram utilizados durante as sessões materiais de papelaria, como bola de isopor, canudo colorido, post-it para demarcação de ponto fixo, lista de pseudopalavras e figuras para nomeação rápida e uma almofada para treino em superfície instável.

Os voluntários foram orientados a repetirem um dos sete exercícios propostos na sessão, em casa, de duas a três vezes ao dia. O exercício selecionado deveria provocar tontura / instabilidade. Apesar do protocolo ser padronizado para todos, as adaptações individuais foram realizadas ,a fim de promover a neuroplasticidade de forma efetiva^(31)^.

### Fotobiomodulação (FBM)

A terapia de FBM foi realizada sempre após a sessão de RV, a aplicação foi executada em uma sala reservada , com o paciente deitado em uma maca, por meio da aplicação de dois aparelhos (de forma binaural) de *LASER* de baixa intensidade DUO de corrente contínua, com potência de 100mW, da marca MMO. Foi utilizado o comprimento de onda de 808 nm (infravermelho), em emissão contínua, no meato acústico externo de forma bilateral e simultânea, com energia de 42 J por aparelho,totalizando 84 J por sessão, sendo selecionado no aparelho, a opção de ilib infravermelho, a fim de realizar uma emissão contínua, durante sete minutos, conforme protocolo padronizado em pesquisa anterior pelos autores do presente estudo.

Foi utilizado a ponteira de bico de laserpuntura *Laser* Duo - Modelo 2.0 para maior aproximação das estruturas alvo , duas vezes por semana. O bico de laserpuntura foi utilizado a fim de aproximar o tecido alvo do feixe de luz, o que permite a estimulação o mais próximo da membrana timpânica. Optou-se ainda pelo uso do infravermelho, pois este apresenta maior profundidade de alcance ao tecido alvo, tendo em vista que a área alvo não terá ponto de contato direto.

A intervenção com FBM foi conduzida com base em um protocolo previamente padronizado pelos autores, em um estudo piloto desenvolvido com 60 orelhas de adultos assintomáticos. Esse estudo teve como objetivo avaliar a segurança do protocolo proposto, por meio da análise da ocorrência de possíveis efeitos adversos, bem como investigar sua eficácia em promover alterações eletrofisiológicas mensuráveis. Embora os dados provenientes desse estudo ainda não tenham sido publicados, os resultados obtidos respaldaram a adoção do referido protocolo na presente investigação, garantindo parâmetros de aplicação seguros e potencialmente eficazes.

### Métodos estatísticos

A análise estatística foi realizada por meio do pacote de *software Statistical Package for the Social Sciences* (SPSS) versão 19.0 (SPSS Inc., Chicago, IL). O teste de *Kolmogorov-Smirnov* e *Shapiro-Wilk* foi usado para avaliar a normalidade da distribuição dos dados (p > 0,05), os quais demonstraram ter uma distribuição normal. A significância estatística foi testada com os modelos lineares globais na caixa de ferramentas do *software* SPSS para medidas repetidas e a comparação pareada para encontrar as diferenças pelo teste de Bonferroni. Além disso, um teste T-*student* foi utilizado para comparar as médias, utilizando o teste T-*student* e análise da mediana por meio do Teste *Mann-Whitney*. A significância clínica foi investigada por meio da mudança mínima detectável (MMD%). A MMD, com 95% de confiança, foi calculada a partir do erro-padrão medida (sEPM) para indicar uma mudança real dos grupos GC e GP.

## RESULTADOS

Ao todo trinta e dois voluntários demonstraram interesse no estudo após a triagem inicial (Figura 2.). Sete deles não puderam ser incluídos porque os SV, zumbido e os fatores associados não se enquadravam nos critérios da pesquisa. Vinte e cinco pacientes foram randomizados e incluídos no estudo. Durante o tratamento ocorreram cinco desistências. Ao total, vinte sujeitos foram incluídos na análise final, dez do grupo controle e dez do grupo pesquisa.

**Figura 2:** Fluxograma dos participantes, conforme o *Consolidated Standards of Reporting Trials* (CONSORT 2010)

Fonte: Elaborada pelo autor

Quanto aos limiares audiométricos, foram incluídos cinco voluntários com audição normal, cinco com perda auditiva sensorioneural de grau leve e dez sujeitos com configuração audiométrica descendente leve, nas frequências agudas. Tanto o GC, como o GP foram formados por nove mulheres e um homem. A Tabela 1 apresenta os dados referentes à faixa etária dos participantes. A idade média foi de 52,50 anos (DP = 8,41) no GC e de 51,70 anos (DP = 10,36) no GP.

**Tabela 1:** Descrição e comparação entre os grupos por meio do parâmetro idade

Variável	Grupo	Mínimo	Máximo	Média	Desvio Padrão	Estatística do Teste	p- valor
Idade	GC	38	60	52,50	8,410	48,500	0,912^1^
	GP	30	60	51,70	10,361		

1 Teste Mann-Whitney;

Legenda: GC = Grupo Controle; GP = Grupo Pesquisa

Fonte: Dados da pesquisa, 2023

Em ambos os grupos, os SV diminuíram após o processo de intervenção terapêutica proposto. A intensidade subjetiva dos SV, medida pela EVA, os escores do DHI físico, emocional, funcional e total, o Índice de Equilíbrio Composto apresentaram melhora nos dois grupos, sem diferença estatística. O parâmetro Análise Sensorial da Função Vestibular mostrou diferença significante nos momentos pré e pós-terapia (p=0,003) apenas no GP.

A área do Limite de Estabilidade média do GC aumentou 1688,19 mm^2^ após as dez sessões de RV, enquanto que no GP houve um aumento de 3516,61 mm^2^ .No entanto, não se verificou diferença estatisticamente significativa. A Tabela 2 demonstra a comparação entre os grupos dos parâmetros de resultado supracitados.

**Tabela 2:** Dados descritivos e de comparação dos grupos nos momentos pré e pós- terapia

**Variável**			**Mínimo**	**Máximo**	**Média**	**Desvio Padrão**	**Estatística do teste**	**p-valor**
**EVA Zumbido**	Grupo Controle (GC)	Pré-Terapia	5	10	7,60	1,955	1,435^1^	0,170
		Pós-Terapia	4	9	6,50	1,434		
	Grupo Pesquisa (GP)	Pré-Terapia	4	10	8,30	2,359	15,000^2^	**0,007** ^*^
		Pós-Terapia	1	10	4,10	3,247		
**EVA Tontura**	Grupo Controle (GC)	Pré-Terapia	2	9	5,9	2,234	3,556^1^	0,003^*^
		Pós-Terapia	2	5	3,10	1,101		
	Grupo Pesquisa (GP)	Pré-Terapia	5	10	7,70	1,829	6,394^1^	0,000^*^
		Pós-Terapia	0	6	2,30	1,947		
**THI Funcional**	Grupo Controle (GC)	Pré-Terapia	8	44	28,40	11,423	0,491^1^	0,629
		Pós-Terapia	8	38	26,00	10,414		
	Grupo Pesquisa (GP)	Pré-Terapia	20	44	32,00	9,238	5,268^1^	0,000^*^
		Pós-Terapia	0	28	11,40	8,222		
**THI Emocional**	Grupo Controle (GC)	Pré-Terapia	6	34	22,40	10,362	0,564^1^	0,580
		Pós-Terapia	4	34	19,80	10,261		
	Grupo Pesquisa (GP)	Pré-Terapia	8	36	22,00	11,700	10,500^2^	0,002*
		Pós-Terapia	0	32	7,20	9,670		
**THI Catastrófico**	Grupo Controle (GC)	Pré-Terapia	8	18	13,20	3,910	1,489^1^	0,157
		Pós-Terapia	0	20	9,80	6,070		
	Grupo Pesquisa (GP)	Pré-Terapia	4	20	13,60	5,948	3,526^1^	0,003*
		Pós-Terapia	0	14	5,40	4,326		
**THI Total**	Grupo Controle (GC)	Pré-Terapia	30	96	64,00	23,438	37,000^2^	0,353
		Pós-Terapia	24	88	55,80	24,284		
	Grupo Pesquisa (GP)	Pré-Terapia	40	100	67,60	25,920	4,094^2^	0,001*
		Pós-Terapia	0	74	24,60	20,764		
**DHI Físico**	Grupo Controle (GC)	Pré-Terapia	12	28	20,80	5,750	4,850^1^	0,000*
		Pós-Terapia	4	18	9,60	4,502		
	Grupo Pesquisa (GP)	Pré-Terapia	16	36	23,80	5,692	7,576^1^	0,000*
		Pós-Terapia	2	14	6,80	4,237		
**DHI Emocional**	Grupo Controle (GC)	Pré-Terapia	8	32	20,00	7,542	4,359^1^	0,001^*^
		Pós-Terapia	4	16	8,20	4,050		
	Grupo Pesquisa (GP)	Pré-Terapia	12	32	21,80	7,208	5,967^1^	0,000*
		Pós-Terapia	0	16	5,80	4,467		
**DHI Funcional**	Grupo Controle (GC)	Pré-Terapia	12	28	21,40	5,892	4,836^1^	0,000^*^
		Pós-Terapia	6	18	10,60	3,893		
	Grupo Pesquisa (GP)	Pré-Terapia	16	30	23,00	5,185	9,000^2^	0,001^*^
		Pós-Terapia	4	26	8,60	7,427		
**DHI Total**	Grupo Controle (GC)	Pré-Terapia	40	82	61,60	15,284	5,878^1^	0,000^*^
		Pós-Terapia	20	48	28,20	9,449		
	Grupo Pesquisa (GP)	Pré-Terapia	44	92	68,60	14,909	1,500^2^	0,000^*^
		Pós-Terapia	8	46	21,20	13,037		
**Limite de Estabilidade**	Grupo Controle (GC)	Pré-Terapia	8710,4	16321,1	12140,060	2633,621	-1,354^1^	0,193
		Pós-Terapia	9879,6	17385,1	13828,250	2935,434		
	Grupo Pesquisa (GP)	Pré-Terapia	7195,7	19269,3	12368,880	3619,764	-1,798^1^	0,091
		Pós-Terapia	10289,5	27289,1	15885,490	5013,693		
**Análise Sensorial da Função Vestibular**	Grupo Controle (GC)	Pré-Terapia	40,0	97,6	84,150	16,746	31,000^2^	0,165
		Pós-Terapia	89,2	98,4	93,370	3,315		
	Grupo Pesquisa (GP)	Pré-Terapia	45,5	95,5	81,540	15,956	12,000^2^	0,003*
		Pós-Terapia	90,1	97,0	94,710	2,2708		
**Índice de Equilíbrio Composto**	Grupo Controle (GC)	Pré-Terapia	50,6	95,7	85,270	14,1144	16,000^2^	0,009*
		Pós-Terapia	81,4	97,5	93,910	4,7864		
	Grupo Pesquisa (GP)	Pré-Terapia	40	100	67,60	25,920	19,000^2^	0,019*
		Pós-Terapia	81,4	97,5	93,910	4,7864		
**AVD Escore Horizontal**	Grupo Controle (GC)	Pré-Terapia	72	90	80,00	5,812	-3,632^1^	0,003*
		Pós-Terapia	80	100	89,20	5,514		
	Grupo Pesquisa (GP)	Pré-Terapia	42	96	71,60	17,859	12,000^2^	0,002*
		Pós-Terapia	80	100	92,20	6,356		
**AVD Escore Vertical**	Grupo Controle (GC)	Pré-Terapia	72	92	81,00	6,055	-3,632^1^	0,002*
		Pós-Terapia	84	100	92,20	6,563		
	Grupo Pesquisa (GP)	Pré-Terapia	48	88	69,00	14,275	-4,100^1^	0,001*
		Pós-Terapia	72	100	90,60	8,592		

1 Teste -T de amostras independentes;

2 Teste Mann-Whitney;

* Dados significantes

Fonte: Dados da pesquisa, 2023

Legenda: GC = Grupo Controle; GP = Grupo Pesquisa; EVA = Escala Visual Analógica; THI = Tinnitus Handicap Inventory; DHI = *Dizziness Handicap Inventory*; AVD = *Acuidade Visual Dinâmica*

Observa-se na Tabela 3 que Acuidade Visual Dinâmica nos escores vertical foi diferente entre os grupos (p=0,031) no momento pré-intervenção, o que demonstra maior déficit do RVO nos movimentos verticais no GP antes do tratamento, porém a diferença não se mantém no pós-intervenção. Com melhora de 11% na média do GC e de 21,6% na média do GP pós-terapia, a Acuidade Visual Dinâmica nos escores horizontal e vertical apresentaram melhora nos dois grupos, sendo o índice de significância ,mais evidente no grupo intervenção (GP).

**Tabela 3:** Dados descritivos e de comparação entre grupos pré e pós-terapia

**Variável**			**Mínimo**	**Máximo**	**Média**	**Desvio Padrão**	**Estatística do teste**	**p-valor**
**EVA Zumbido**	Pré- Terapia	GC	5	10	7,60	1,955	40,000^1^	0,481
		GP	4	10	8,30	2,359		
	Pós- Terapia	GC	4	9	6,50	1,434	0,213^2^	**0,046** ^*^
		GP	1	10	4,10	3,247		
**EVA Tontura**	Pré- Terapia	GC	2	9	5,9	2,234	-1,972^2^	0,065
		GP	5	10	7,70	1,829		
	Pós- Terapia	GC	2	5	3,10	1,101	1,131^1^	0,277
		GP	0	6	2,30	1,947		
**THI Funcional**	Pré- Terapia	GC	8	44	28,40	11,423	-0,775^2^	0,448
		GP	20	44	32,00	9,238		
	Pós- Terapia	GC	8	38	26,00	10,414	3,480^2^	0,003^*^
		GP	0	28	11,40	8,222		
**THI Emocional**	Pré- Terapia	GC	6	34	22,40	10,362	48,000^1^	0,912
		GP	8	36	22,00	11,700		
	Pós- Terapia	GC	4	34	19,80	10,261	12,000^1^	0,002^*^
		GP	0	32	7,20	9,670		
**THI Catastrófico**	Pré- Terapia	GC	8	18	13,20	3,910	-0,178^2^	0,861
		GP	4	20	13,60	5,948		
	Pós- Terapia	GC	0	20	9,80	6,070	1,867^2^	0,080
		GP	0	14	5,40	4,326		
**THI Total**	Pré- Terapia	GC	30	96	64,00	23,438	-0,326^2^	0,748
		GP	40	100	67,60	25,920		
	Pós- Terapia	GC	24	88	55,80	24,284	-4,326	0,000^*^
		GP	0	74	24,60	20,764		
**DHI Físico**	Pré- Terapia	GC	12	28	20,80	5,750	-0,172^2^	0,256
		GP	16	36	23,80	5,692		
	Pós- Terapia	GC	4	18	9,60	4,502	1,432^2^	0,169
		GP	2	14	6,80	4,237		
**DHI Emocional**	Pré- Terapia	GC	8	32	20,00	7,542	-0,546^2^	0,592
		GP	12	32	21,80	7,208		
	Pós- Terapia	GC	4	16	8,20	4,050	30,500^1^	0,143
		GP	0	16	5,80	4,467		
**DHI Funcional**	Pré- Terapia	GC	12	28	21,40	5,892	-0,645^2^	0,527
		GP	16	30	23,00	5,185		
	Pós- Terapia	GC	6	18	10,60	3,893	25,500^1^	0,063
		GP	4	26	8,60	7,427		
**DHI Total**	Pré- Terapia	GC	40	82	61,60	15,284	-1,037^2^	0,314
		GP	44	92	68,60	14,909		
	Pós- Terapia	GC	20	48	28,20	9,449	3,480^2^	0,159
		GP	8	46	21,20	13,037		
**Limite de Estabilidade**	Pré- Terapia	GC	8710,4	16321,1	12140,060	2633,6216	-0,162^2^	0,874
		GP	7195,7	19269,3	12368,880	3619,7642		
	Pós- Terapia	GC	9879,6	17385,1	13828,250	2935,4343	39,000^1^	0,436
		GP	10289,5	27289,1	15885,490	5013,6937		
**Análise Sensorial da Função Vestibular**	Pré- Terapia	GC	40,0	97,6	84,150	16,746	43,000^1^	0,631
		GP	45,5	95,5	81,540	15,956		
	Pós- Terapia	GC	89,2	98,4	93,370	3,315	-1,055^2^	0,307
		GP	90,1	97,0	94,710	2,2708		
**Índice de Equilíbrio Composto**	Pré- Terapia	GC	50,6	95,7	85,270	14,114	46,000^1^	0,796
		GP	40	100	67,60	25,920		
	Pós- Terapia	GC	81,4	97,5	93,910	4,7864	46,500^1^	0,791
		GP	81,4	97,5	93,910	4,7864		
**AVD Escore Horizontal**	Pré- Terapia	GC	72	90	80,00	5,812	40,000^1^	0,481
		GP	42	96	71,60	17,859		
	Pós- Terapia	GC	80	100	89,20	5,514	-1,127^2^	0,275
		GP	80	100	92,20	6,356		
**AVD Escore Vertical**	Pré- Terapia	GC	72	92	81,00	6,055	2,447^2^	0,031*
		GP	48	88	69,00	14,275		
	Pós- Terapia	GC	84	100	92,20	6,563	0,468^2^	0,646
		GP	72	100	90,60	8,592		

1 Teste Mann-Whitney;

2 Teste -T de amostras independentes;

* Dados significantes

Legenda: GC = Grupo Controle; GP = Grupo Pesquisa; EVA = Escala Visual Analógica; THI = Tinnitus Handicap Inventory; DHI = *Dizziness Handicap Inventory*; AVD = *Acuidade Visual Dinâmica*

Fonte: Dados da pesquisa, 2023

Em contrapartida, em relação aos parâmetros avaliativos do zumbido, apenas o GP demonstrou melhora estatisticamente significante nos momentos pré e pós-terapia , assim como na comparação entre os grupos no momento pós-terapia (Tabela 3); EVA Zumbido (p=0,007), THI Funcional (p=0,000), THI Emocional (p=0,002), THI Total (p=0,003). Não houve diferença significativa no THI Catastrófico (p=0,080).

## DISCUSSÃO

No presente estudo, a média de idade foi de 52,50 anos no grupo controle (GC) e 51,70 anos no grupo de pesquisa (GP), sendo a perda auditiva neurossensorial de configuração descendente leve nas frequências agudas, a mais frequente. Esse achado é compatível com os padrões audiométricos descritos na literatura para a presbiacusia, caracterizada por uma perda auditiva progressiva, bilateral e simétrica, com maior comprometimento das frequências agudas^(10)^.

Além das alterações auditivas, as disfunções vestibulares tornam-se mais proeminentes com o avanço da idade, o que sugere, uma possível associação entre a degeneração dos sistemas auditivo e vestibular ao longo do processo de envelhecimento^(32)^. Estudos apontam que essas alterações podem estar relacionadas a mecanismos fisiopatológicos comuns, como a degeneração neuronal no nervo vestibulococlear e mudanças na hidrodinâmica dos fluidos da orelha interna, que afetam tanto a cóclea quanto os canais semicirculares^(33)^.

Considerando, portanto, que tanto o zumbido quanto os sintomas vestibulares são mais prevalentes em indivíduos mais velhos^(14)^, é possível que a fisiopatologia do envelhecimento tenha desempenhado um papel relevante nos achados da pesquisa. sendo caracterizada por alterações na homeostase do cálcio e perda da função neural periférica^(34,35)^.

Estudo^(36)^ em roedores demonstrou que esta redução das proteínas ligantes de cálcio está associada ao aumento do limiar eletrofisiológico do Potencial Evocado Auditivo de Tronco Encefálico (PEATE ) e redução da amplitude das emissões otoacústicas por produto de distorção em roedores mais velhos.

Outro estudo^(37)^, de revisão sistemática identificou que, dentre os mecanismos moleculares desencadeados após a FBM, um deles é o aumento da produção de ATP, o que induz o influxo de cálcio intracelular, e permite regular os níveis tóxicos do conteúdo intracelular de cálcio. Estudo^(20)^ *in vitro* e *in vivo* de células da cóclea demonstrou efeitos positivos da FBM, como redução dos níveis de estresse oxidativo, recuperação histológica das células após o tratamento com aminoglicosídeos e melhor recuperação de limiares eletrofisiológico após trauma acústico induzido. Esses achados reforçam a necessidade de investigações adicionais para esclarecer se a resposta à FBM pode ser modulada por fatores etários e se essa terapia apresenta maior benefício em populações com comprometimentos metabólicos relacionados ao envelhecimento.

Revisão sistemática com metanálise^(19)^ sugere que pacientes com zumbido decorrente de lesão por trauma acústico ou degeneração do nervo auditivo podem apresentar maior benefício com a FBM em comparação a outras possíveis etiologias do zumbido. Esse achado pode explicar os resultados positivos observados no presente estudo.

O presente estudo demonstrou diferença entre os grupos, quanto aos parâmetros de zumbido (EVA e THI), que corrobora com pesquisas dentro da clínica otoneurológica que demonstram desfechos positivos do uso da FBM em pacientes com zumbido^(38-42)^.

Mollasadeghi et al.^(37)^ investigou pacientes com perda auditiva neurossensorial bilateral submetidos a 20 sessões de fotobiomodulação (FBM) com parâmetros distintos (650 nm, 5 mW, estimulação na mastoide). Da mesma forma, outra pesquisa^(39)^ utilizou um comprimento de onda infravermelho (830 nm) com potência de 100 mW, semelhante ao protocolo adotado no presente estudo, mas com uma energia irradiada de 120 J por sessão. Essa pesquisa encontrou melhoras na intensidade e na duração do zumbido, porém sem diferenças estatísticas para os escores da EVA e do THI. Além disso, foi observado que os participantes do grupo que não apresentaram melhora possuíam limiares auditivos significativamente piores do que aqueles que obtiveram benefícios.

Estudo^(40)^ recente reforça que o efeito positivo da FBM é dependente das diferentes configurações de aplicação. O referido ensaio clínico^(40)^ indicou que o grupo com melhores desfechos no THI, utilizou um protótipo de laser vermelho transmeatal (660 nm, 100 mW, 180 J por sessão) com aplicação bilateral, apresentando resultados superiores em comparação com os participantes que receberam uma dose menor de energia do mesmo equipamento (72 J por sessão). Vale ressaltar que o comprimento de onda influencia a penetração tecidual, sendo que 660 nm tende a atingir camadas mais superficiais, enquanto 808 nm apresenta maior profundidade de absorção^(18-20,37)^. Essa diferença pode explicar os efeitos positivos observados no presente estudo, que adotou uma configuração de 84 J por sessão, porém infravermelho, com uma ponteira mais próxima à membrana timpânica.

Outro estudo^(41)^ analisou o impacto da FBM com um comprimento de onda infravermelho (830 nm, 67 mW, 80,4 J/cm^2^) em doze sessões distribuídas ao longo de quatro semanas. Os resultados apontaram diferenças estatisticamente significativas na EVA e no THI quando comparados ao grupo controle. Estudo^(42)^ que utilizou equipamento com potência de 5mW, comprimento de onda de 650mm, durante 20 minutos (6 J por sessão ) em mergulhadores, com audição normal e presença de zumbido apresentaram resultados significativos após 40 e 60 sessões, porém os autores utilizaram como critério para avaliar o desfecho, uma escala não-validada.

Apesar dos achados positivos relatados anteriormente, alguns estudos apresentaram resultados divergentes, sem distinção significativa entre o grupo de intervenção e o grupo placebo^(43-46)^. Dentre esses, apenas um estudo^(43)^ utilizou um equipamento de laser com potência de 100 mW, comprimento de onda de 606 nm, aplicado de forma transmeatal, com dose de 4 J por sessão, sem evidenciar diferenças estatisticamente significativas entre os grupos.

Além disso, outras pesquisas^(44-46)^ investigaram a FBM transmeatal utilizando aparelhos de potência inferior a 7 mW e comprimento de onda na faixa do vermelho, mas também não observaram efeitos positivos no tratamento do zumbido idiopático. Esses achados sugerem que fatores como potência e parâmetros de aplicação podem influenciar os desfechos da terapêutica.

Quanto aos achados dos SV, o presente estudo demonstrou a efetividade do programa de RV elaborado pelos autores, evidenciada pela redução significativa dos escores totais do DHI nas fases pós-tratamento em ambos os grupos. Esses achados corroboram a literatura^(2,3,8,9)^ que aponta a RV como uma abordagem eficaz nas hipofunções vestibulares, com evidência de moderada a forte, reforçando os resultados obtidos neste estudo. No entanto, a associação com a FBM demonstrou potencial adicional na melhora de variáveis como a análise sensorial da função vestibular e o limite de estabilidade, o que sugere que a fotobiomodulação pode atuar de forma sinérgica, promovendo efeitos neuroplásticos e reparadores no sistema vestibular

Em relação aos parâmetros da posturografia, observa-se melhora estatisticamente significante da Análise Sensorial da Função Vestibular no GP. Embora tenha ocorrido um aumento na média pós-intervenção do Limite de Estabilidade (LE) em ambos os grupos, este também foi maior no GP. O LE equivale à capacidade que o indivíduo apresenta para deslocar voluntariamente o seu centro de massa, com precisão e velocidade, sem alterar sua base de suporte^(28,46)^. Este parâmetro está proporcionalmente relacionado ao risco de queda. Estudo^(46)^ demonstra que o aumento do LE está correlacionado com a melhora da análise sensorial da função vestibular , o que corrobora com nossos achados. Não obtivemos diferenças entre o Índice de Equilíbrio Composto.

Quanto à Acuidade visual dinâmica tanto o GP, como o GC apresentaram melhora estatisticamente significante entre os momentos pré e pós-terapia. Porém o grupo que realizou a FBM verdadeira apresentou aumento significantemente maior na média dos escores horizontal e vertical em relação ao GC. A medida da acuidade visual durante o movimento da cabeça tem sido utilizada para avaliar a funcionalidade do impacto da hipofunção vestibular, alterações na acuidade visual impactam nas atividades diárias dos pacientes, como dirigir, ler, assistir televisão^(27)^. O teste vem sendo relatado na literatura como confiável em termos de sensibilidade e especificidade, sendo capaz de distinguir indivíduos normais e pacientes com perda vestibular^(27)^.

Não foram encontrados ensaios clínicos, com FBM transmeatal bilateral, com comprimento de onda infravermelho em pacientes com zumbido e nem em FBM para pacientes com alterações vestibulares independente do parâmetro de irradiação.

Até onde sabemos, este estudo é o primeiro ensaio clínico prospectivo randomizado controlado que demonstra o efeito benéfico da FBM associada à RV em indivíduos com SV e zumbido. A limitação deste estudo é que o pequeno número de voluntários pode não ser suficiente para tirar conclusões sólidas sobre os efeitos clínicos e apesar da doença de base não ter sido considerada na análise do estudo, os sinais clínicos foram delimitados nos critérios de inclusão e exclusão, o que possibilitou grupos com características e sintomas homogêneos.

Não encontramos melhora dos parâmetros do sintoma zumbido, no grupo que realizou a FBM placebo, portanto a RV não foi capaz de intervir nesse sintoma, também não encontramos ensaios clínicos utilizando a RV para tratamento do Zumbido. Mais estudos longitudinais e prospectivos são necessários para avaliar os resultados em longo prazo dos efeitos da FBM associado a RV em SV, assim como a FBM associada à intervenções com comprovações científicas no zumbido, utilizando um tamanho de amostra maior, com acompanhamento em longo prazo.

## CONCLUSÃO

Os resultados deste estudo demonstram que a fotobiomodulação (FBM) associada à Reabilitação Vestibular (RV) apresentou efeitos significativos na redução do incômodo do zumbido , conforme avaliado por meio da EVA e THI, assim como na Análise Sensorial da Função Vestibular , analisada pela posturografia , quando comparado ao grupo placebo . Houve ainda melhora dos parâmetros da Acuidade Visual Dinâmica e Limite de Estabilidade, em relação ao Grupo Controle, porém sem efeito estatístico. Não foram observadas diferenças entre os grupos, em relação a EVA Tontura, DHI e Índice de Equilíbrio Composto.Também não foram encontrados efeitos positivos da RV para o sintoma zumbido.

## Apêndice A Protocolo de reabilitação vestibular

**Table:**

**1º Semana, sessões 1 e 2**
1. Olhar para cima e para baixo mirando para dois pontos fixos, devagar e depois rapidamente (em pé) (2 minutos)
2. Olhar para a direita e para a esquerda mirando para dois pontos fixos, devagar e depois rapidamente (em pé) (2 minutos)
3. Aproximar e afastar canudo multicolorido ( convergência visual), olhando para ele (em pé) (lentamente e depois rapidamente) (2 minutos)
4. Jogar uma bola de uma mão para outra, acompanhando-a com os olhos. (30 vezes).
5. Colocar um objeto no chão. Apanhá-lo e elevá-lo acima da cabeça e colocá-lo no chão novamente (olhando para o objeto o tempo todo). (3 séries de 10 repetições)
6. Sentar, depois levantar e girar para um lado, sentar novamente, levantar de novo e agora girar para o outro lado. (15 vezes)
7. Gire a cabeça 45 graus de um lado para o outro, sem parar, mantendo o olhar focalizando em “X “num cartão à sua frente, durante um a dois minutos, lentamente na primeira sessão e rapidamente na segunda. (2 minutos)
**2º Semana, sessões 3 e 4**
1. Sentado, virar a cabeça para direita, olhar para cima e para baixo e para trás, acompanhando estes movimentos oculares, com a movimentação concomitante da cabeça. (10 vezes). Sentado, virar a cabeça para esquerda, olhar para cima e para baixo e para trás, acompanhando estes movimentos oculares, com a movimentação concomitante da cabeça. (10 vezes) (Realizar duas séries)
2. Gire a cabeça 45 graus de um lado para o outro, com uma breve pausa com a cabeça centralizada, mantendo o olhar focalizando em “X “num cartão à sua frente, durante um a dois minutos, começando lentamente e depois rapidamente (em pé). (2 minutos)
3. Repetir o exercício anterior, no sentido vertical (em pé). (2 minutos)
4. Mover a cabeça em flexão e extensão com os olhos abertos, no plano vertical, como se expressasse um “sim” com a cabeça, mantendo o olhar fixo (em pé) (lentamente e depois rapidamente olhando para um ponto fixo na parede) (3 minutos), ao sentir tontura parar por 10 segundos e recomeçar, até atingir os 3 minutos de exercício.
5. Mover a cabeça para a direita e para a esquerda com os olhos abertos, movimentos cefálicos no plano horizontal, expressar um “não”, mantendo o olhar fixo (em pé) (lentamente e depois rapidamente), ao sentir tontura parar por 10 segundos e recomeçar, até atingir os 3 minutos de exercício.
6. Dar um passo e fazendo rotação cervical para a direita e para a esquerda, andando de frente e de costas. (3 minutos)
7. Ficar em pé, com os pés o mais afastados possível, mirando em um alvo a sua frente. Estreitar progressivamente a base de sustentação colocando sucessivamente pés juntos, um pé parcialmente adiante do outro, com os braços abertos, depois ao longo do corpo e por fim cruzados no peito. (15 vezes)
**3º Semana, sessões 5 e 6**
1. Ficar em pé, com os pés o mais afastados possível, com a cabeça fletida 30 graus para a frente, com os braços abertos, depois ao longo do corpo e por fim cruzados no peito. (15 vezes)
2. Ficar em pé, com os pés o mais afastados possível, com a cabeça flexionada 30 graus para trás, com os braços abertos, depois ao longo do corpo e por fim cruzados no peito. (15 vezes)
3. Mover a cabeça em flexão e extensão com os olhos abertos (andando para frente e para trás ( quando possível) (rapidamente olhando para um ponto fixo na parede) (3 minutos), ao sentir tontura parar por 10 segundos e recomeçar, até atingir os 3 minutos de exercício.
4. Mover a cabeça para a direita e para a esquerda com os olhos abertos (andando para frente e para trás( quando possível) (rapidamente olhando para um ponto fixo na parede), ao sentir tontura parar por 10 segundos e recomeçar, até atingir os 3 minutos de exercício.
5. Andar com o pé na frente do outro com os olhos abertos, para frente.( Exercício poderá ser realizado com o apoio de mão , caso necessário). (3 minutos)
6. Andar e fazer rotação cervical para a direita e para a esquerda, andando de frente e de costas ( quando possível), sem realizar pausas entre as passadas. (3 minutos)
7. Movimentar um cartão e a cabeça em direções opostas, no plano horizontal ,sem parar, mantendo foco em uma lista de palavras (2 minutos).
**4º Semana, sessões 7 e 8**
1. Movimentar um cartão e a cabeça em direções opostas, sem parar, mantendo foco em uma lista de pseudo-palavras (2 minutos), repetir no plano vertical (2 minutos).
2. Marchar no lugar, em cima de um travesseiro, fixando o olhar a frente, 10 segundos com os olhos fechados e 10 segundos com os olhos abertos. (10 vezes) (exercício poderá ser realizado com apoio de mão)
3. Em cima de um travesseiro, movimentar a cabeça, fletindo-a de um lado para o outro, olhando para um ponto fixo, 10 segundos com os olhos abertos e 10 segundos com os olhos fechados. (exercício poderá ser realizado com apoio de mão).
4. Ficar na posição de pé na frente do outro, com os olhos abertos, em cima do travesseiro, 1 minuto em cada posição de pé (exercício com apoio do terapeuta)
5. Andar com um pé na frente do outro, com os olhos abertos, para frente, movimentando os olhos nas laterais direita e esquerda (3 minutos).
6. Marchar (caminhando) e formando uma figura similar a um 8, olhando para um cartão com um x. (2 minutos)
7. Marchar dando cinco passos para frente e girar 360 graus para a direita, posteriormente dar mais cinco passos (marchando) e virar 360 graus para a esquerda. (2 minutos)
**5º Semana, sessões 9 e 10**
1. Marchar dando cinco passos para frente e girar 360 graus para a direita, posteriormente dar mais cinco passos (marchando) e virar 360 graus para a esquerda (de olhos fechados). (2 minutos).
2. Marchar no lugar, em cima de um travesseiro, mover a cabeça para a direita e para a esquerda com os olhos abertos, movimentos cefálicos no plano horizontal, expressar um “não”, mantendo o olhar fixo em um ponto. (2 minutos)
3. Marchar no lugar, em cima de um travesseiro, mover a cabeça no plano vertical, como se o paciente expressasse um “sim” com a cabeça, mantendo o olhar fixo o olhar fixo em um ponto. (2 minutos)
4. Andar com o pé na frente do outro, com os olhos abertos, para frente, movimentando o cabeça nas laterais direita e esquerda (2 minutos). (exercício poderá ser realizado com apoio de mão).
5. Marchar dando voltas circulares em uma cadeira enquanto olha para pontos fixos colocados a direita e a esquerda 10x em cada sentido. (2 séries)
6. Andar marchando e mover a cabeça para a direita e para a esquerda com os olhos abertos, movimentos cefálicos no plano horizontal, expressar um “não”. (2 minutos, com olhos abertos). (exercício poderá ser realizado com apoio de mão, próximo a parede).
7. Marchar e andando para frente, movimentar a cabeça, fletindo a de um lado para o outro, enquanto realiza nomeação rápida de figuras. (3 minutos)

Fonte: Autores (2026), adaptado de Cawthorne e Cooksey, AOOI, Herdman, Davis e O’Leary citados em Morozetti et al.^(29)^ e UNIFESP^(30)^
